# Nanoparticle Surface Functionalization: How to Improve Biocompatibility and Cellular Internalization

**DOI:** 10.3389/fmolb.2020.587012

**Published:** 2020-11-26

**Authors:** Gennaro Sanità, Barbara Carrese, Annalisa Lamberti

**Affiliations:** Department of Molecular Medicine and Medical Biotechnology, University of Naples Federico II, Naples, Italy

**Keywords:** nanoparticles, toxicity, biocompatibility, uptake, functionalization

## Abstract

The use of nanoparticles (NP) in diagnosis and treatment of many human diseases, including cancer, is of increasing interest. However, cytotoxic effects of NPs on cells and the uptake efficiency significantly limit their use in clinical practice. The physico-chemical properties of NPs including surface composition, superficial charge, size and shape are considered the key factors that affect the biocompatibility and uptake efficiency of these nanoplatforms. Thanks to the possibility of modifying physico-chemical properties of NPs, it is possible to improve their biocompatibility and uptake efficiency through the functionalization of the NP surface. In this review, we summarize some of the most recent studies in which NP surface modification enhances biocompatibility and uptake. Furthermore, the most used techniques used to assess biocompatibility and uptake are also reported.

## Introduction

Nanoparticles (NPs) are ultrafine particles with a size between 10 and 500 nm composed of different organic and/or inorganic materials (Jeevanandam et al., 2018). These particles have been widely studied because of their unique properties and find suitable application in a great number of biomedical fields like biomolecule detection, vaccines, regenerative medicine, and tissue engineering, gene and drug delivery, cancer therapy, high accuracy diagnosis, and theranostics (Rudramurthy and Swamy, 2018).

When NPs are used in biomedical applications two very important characteristics must be considered: toxicity and cellular uptake (Rees et al., 2019; Santos-Rasera et al., 2019; Zhang C. et al., 2019). In fact, the biocompatibility of NPs is one of the most critical characteristics of nano-platforms to be suitable for biomedical purposes (Elmowafy et al., 2019; Liu et al., 2020), and the NPs capability to be internalized by target cells, compared to not-target cells, is a very important goal (Emami et al., 2019; Khan et al., 2019; Khanna et al., 2019).

**FIGURE 1:**
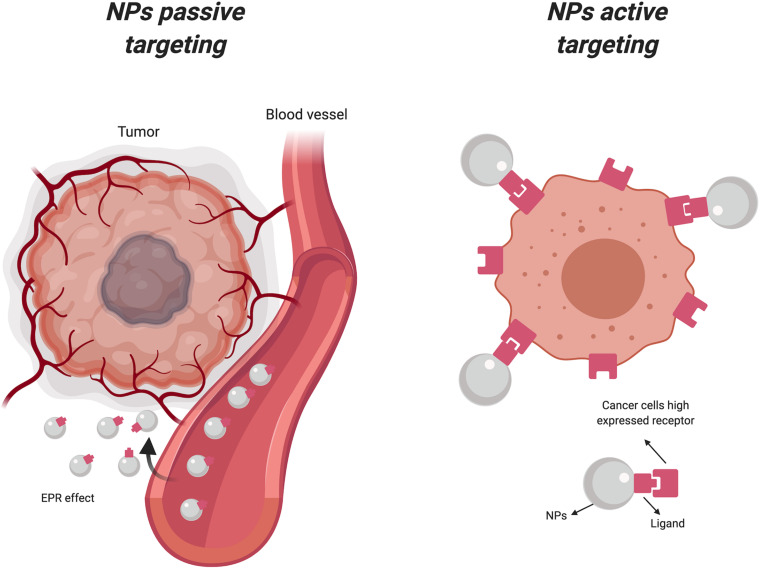
Active and passive uptake of nanoparticles. NPs, Nanoparticles; EPR, Enhanced permeability retention (Created with BioRender.com).

The use of NPs has grown exponentially in the last 15 years especially for cancer treatment, because of their capability to perform high precision tasks, such as the delivery of drugs and imaging contrast agents (CAs) directly to tumor cells (Panebianco et al., 2019; Zhang X. et al., 2019), by using a large number of molecular targets (Yoo et al., 2019). In particular, the accumulation of nanocarriers in cancer cells can occur through two different mechanisms: passive and active targeting. In passive targeting, NPs accumulate in the proximity of the tumor site as result of the altered permeability of tumor blood vessels. This phenomenon, known as the enhanced permeability retention (EPR) effect, permits the passive accumulation of NPs to solid tumors and/or metastatic sites, simply through their particular physical properties including size, shape, and superficial charge (Maeda, 2015). Active targeting exploits the biofunctionalization of the NPs surface by using ligands with a strong affinity and specificity for overexpressed receptors and molecules on the tumor cells (Bertrand et al., 2014; Yoo et al., 2019) or secreted proteins in the tumor microenvironment (TME) (Huai et al., 2019). Active and passive targeting are phenomena often occur simultaneously, and one does not preclude the other (Figure 1).

The use of NPs for active targeting of drugs and/or contrast agents is based on the interaction of properly modified NPs surface with a molecular target overexpressed by cells and tissues. The molecules used to modify the NPs surface include small proteins, peptides, antibodies, aptamers, and oligosaccharides (Large et al., 2019; Yoo et al., 2019). Furthermore, the biochemical modification of the NPs surface with these specific targeting ligands is often necessary to reduce toxicity (as widely applied for silver NPs) and to increase their stability in biological fluids (Guerrini et al., 2018; Borowik et al., 2019). An example is represented by the use of human albumin, whose presence on the NPs surface reduces toxicity and achieves active targeting at the same time (Li D. et al., 2018; Sanità et al., 2020). The chemical modification of the NPs surface is a step-by-step process that requires chemical-physical and biological characterization for each step. Transmission electron microscopy (TEM), scanning electron microscopy (SEM), Fourier transform infrared spectroscopy (FTIR), dynamic light scattering (DLS), and ζ-potential analysis are some of the most useful and simple techniques used to study shape, size, chemical composition, and superficial charge of NPs (Rea et al., 2014; Silvestri et al., 2019). In contrast, the biological evaluation of NPs requires a more complex analysis with the assessment of numerous variables depending on the experimental model, conditions, and other specific parameters (Terracciano et al., 2015; Barot et al., 2020; Sanità et al., 2020). In conclusion, it is crucial to consider the effects of the NPs surface modification on stability, uptake, and biocompatibility to achieve an efficient nanoplatform for biomedical applications.

The focus of this review is to summarize the most used techniques to evaluate NPs toxicity and uptake and to describe the most useful strategies to avoid these issues through the modification of the NPs surface.

## The Role of Nanoparticles in Cancer Disease

NPs can be applied to cancer for two main purposes: diagnosis and therapy (Borkowska et al., 2020; Tammaro et al., 2020). The usage of nanoplatforms for diagnosis can be classified by two different approaches: biomolecule detection and imaging techniques. Biomolecule detection is based on the identification of tumor markers that are often present in a very small amount in biological samples (Marrugo-Ramírez et al., 2018). The possibility to modify NPs by specific ligands with high affinity for these markers can improve the identification and quantification of desired compounds (Kiplagat et al., 2019).

Medical imaging techniques have evolved very quickly over the past 20 years, overall in cancer disease, to identify very little tumor masses for an early diagnosis (Kumar et al., 2019; Li et al., 2019c; Vaidya et al., 2019). This evolution has occurred through the combined use of classical imaging techniques (like MRI, PET and SPECT) with NPs (Forte et al., 2019; Song et al., 2020) and advancing toward the use of innovative imaging techniques like photoacoustic (PA), surface-enhanced Raman scattering (SERS), and near infrared light up-conversion (NIR-Up) (Silvestri et al., 2019; Ge et al., 2020; Kim et al., 2020). All these techniques are very useful in combination with NPs-mediated delivery in order to accumulate CAs in the tissues of interest.

For cancer therapy, there are currently numerous agents available depending on the disease stage, location, type of tissue, age, and conditions of the patient (Reece-Mills et al., 2016; Cohen, 2017). The use of classical chemotherapeutic compounds, radiation, and surgical interventions are still useful and widely used for treating tumors, but the side effects are often a serious concern (Oun et al., 2018; Schirrmacher, 2019). Furthermore, the recurrence of cancer due to the incomplete removal of malignant cells is a major reason of poor prognosis (Chihara et al., 2017; Corrado et al., 2017). The use of NPs for an early and precise identification of cancer cells can help to avoid delayed diagnosis, and the specific delivery of a limited amounts of drug directly to cancer cells can effectively reduce chemotherapy-related side effects (Parvanian et al., 2017; Zhao et al., 2018; Chen Y. et al., 2019). At the same time, the usage of NPs can be useful to bypass the drug resistance of some tumors, like melanoma (Naves et al., 2017; Avagliano et al., 2019). In this scenario, the surface modification of NPs represents an important strategy to successful develop specific and biocompatible nano-platforms for precise and sensitive therapy and/or diagnosis.

## Methods for NPs Surface Modification and Uptake and Biocompatibility Evaluation

The surface modification of nanoparticles is a powerful methodology to fix or attenuate issues related to NPs toxicity and uptake, since both phenomena are closely related to the NPs surface composition.

### Surface Modifications: Covalent and Non-covalent Bonds

The surface functionalization of NPs involves a process that aims to improve and/or add properties useful for the use of NPs in medical applications. Different types of nanomaterials have characteristic chemical properties and functional groups exposed on their surface to be used in the first steps of functionalization. Generally, the first phase of the surface modification is based on the use of homo- or hetero-bifunctional cross linkers to the aim to add an organic functional group (R-NH_2_, R-COOH, etc.), useful to bind biological molecules. For silica NPs, the most used linkers are aminosilanes that introduce an amino group on the NPs surface for the next bio-conjugation (Jung et al., 2012; Rea et al., 2014). Noble metals, like gold, can be functionalized by using crosslinkers with -SH or -NH_2_ groups able to react with the metal and to produce a covalent bond. These bi-functional linkers, such as thio-carboxylic acids, have at the other end functional groups to use for binding ligands (Banihashem et al., 2020). Metal oxides can be easily modified by using a ligand exchange strategy based on the substitution of the original surfaces with functional groups such as diol, amine, carboxylic acid, and thiol useful for the next steps (Korpany et al., 2016). The carbon-based nanomaterials contain a significant fraction of *sp*^2^ hybridized carbon atoms that can be exploited to generate functional groups. Through the oxidation it’s possible to generate -COOH, -OH, and -C = O on the NPs surface (Chen et al., 2016); through halogenation, it’s possible to obtained halogenated carbon that can be further modified, for example by reaction with the amine group (Poh et al., 2013); through cycloaddition it’s possible to insert different type of functional groups (Kiang Chua and Pumera, 2013). Table 1 summarizes types of nanomaterials, their chemical groups and/or composition, and the suitable compounds or processes that can be used for surface modification by using crosslinkers.

**TABLE 1:** Resume of the most common strategies used to modify NPs surface related to the nanomaterials.

Material	Usable functional/chemical groups	Example of chemical compounds/processes suitable for surface modification
Silica	−SiOH	X-Si(OC_2_H_5_)_3_
Noble metals	−Au; −Ag (plasmonic metals)	X-SH, X-NH_2_
Metal oxide	MO_x_	X-COOH; X-(OH)_n_; X-NH_2_ (adsorption)
Carbon based	sp^2^ hybridize carbon	Oxidation; halogenation, cycloaddition …

The modification of the NPs surface can be achieved using two different approaches: non-covalent and covalent conjugation. The non-covalent strategy is based on a large number of weak interactions (electrostatic, ionic, van der Walls and hydrophobic interactions, absorption, hydrogen bonds) and it is specially used with metallic and silica NPs (Cheng et al., 2011; Nell et al., 2016; Yue et al., 2019). Non-covalent bonds have the advantage of being relatively simple and do not affect the structure of the used molecules and their interaction with biological targets. Conversely, non-covalent modifications can be easily influenced by different variables, such as pH and ionic strength (Nobs et al., 2004).

The covalent bond strategy can be obtained by using many alternative approaches, depending on the composition of the NPs (Abánades Lázaro et al., 2017; Oriana et al., 2018; Sakaguchi et al., 2019). Moreover, this strategy allows modifications at several levels using sequential functionalization (Gong et al., 2015; Tian et al., 2015; Świetek et al., 2019). This methodology can be exploited to achieve structures with multiple functions (Chen and He, 2015; Luo et al., 2016), such as diagnosis and therapy to implement the theranostic approach (Shen et al., 2017; Jung et al., 2018). Usually, the covalent bond of ligands to the NPs surface can be achieved using various linker molecules. An example is PEG, that can be synthesized with specific functional groups at the ends and used as homobifunctional or heterobifunctional linkers to perform a wide range of functionalization processes. Pagels et al. (2020) showed how the production of specific heterobifunctional PEG molecules is still an active research field and how this molecule can be very useful to design efficient nano-platforms for medical applications. Thanks to its polymeric nature, PEG can be also used as a spacer for high molecular weight molecules in order to space them across the surface of the NPs and to reduce steric hindrance of bonded ligands, allowing bioconjugation at high density (Chen S. et al., 2017). Generally, non-covalent interactions are used to load nanoparticles with molecules that must be released in target cells, such as drugs or RNAi, while covalent bonds are employed to bind ligands useful to achieve targeting and/or to reduce he toxicity of NPs. Recently, the use of sensitive bonds, such as pH-sensitivity or heat-sensitivity, to develop nanoplatforms for a controlled drug-release has been widely explored (Tai et al., 2009; Lv et al., 2016; Deirram et al., 2019). In particular, the tumor microenvironment is very acidic when compared to the normal microenvironment. pH-sensitive nano-platforms can been designed for controlled drug release specifically triggered by the acidity of the tumor environment. In a recent work, a cationic polymer PBAE pH-sensitive was used to cover liposome NPs loaded with doxorubicin. Furthermore, the NPs surface was modified with hyaluronic acid (HA) to perform active targeting via CD44. *In vivo* experiments confirmed the results obtained *in vitro*, showing that the DOX-loaded NPs inhibited the growth of tumor more efficiently compared to free drug and also reduced side-effects (Men et al., 2020). Another example of pH-sensitive NPs is reported by Taleb et al. (2019). In this work, mesoporous silicon nanoparticles were functionalized with amine conjugated phenylboronic acid linked to dopamine with a pH-sensitive covalent bond. In a weakly acidic environment, as like the tumor environment, the nanoparticles released dopamine owing to the hydrolysis of boronic-ester bond between the two molecules. This intelligent release resulted in an inhibition of vascular endothelial cell migration and tubule formation.

### Evaluation of NPs Uptake

Several methods have been used to evaluate the internalization of nanoparticles in a specific cellular context that can be distinguished as label-free and label-based techniques. Among the label free methods, the most commonly used are transmission electron microscopy (TEM), scansion electron microscopy (SEM), and Raman microscopy. These procedures, unlike label-based ones, have the advantage of not requiring the use of fluorophores that could affect the NPs size or chemical properties. Furthermore, using labeled-NPs complicates the discrimination of internalized NPs or those attached to the cell membrane. Conversely, TEM and SEM imaging, despite offering high-resolution down to the cellular organelle scale, are relatively expensive and time-consuming. Furthermore, TEM and SEM, unlike Raman microscopy, are destructive imaging approaches.

TEM analysis is widely used to study nanoparticle uptake and cellular localization. Rio-Echevarria et al. (2019) showed a better affinity of the epoxy-coated SiO_2_ nanoparticles for the cell membrane, compared to bare NPs, in A549 cells and human monocytes. Furthermore, TEM allowed to identify the nanoparticles in endosomes. Youhannayee et al. (2019) used TEM analysis to evaluate the uptake of bare and APTES-coated iron oxide nanoparticles in PC3 (prostate cancer epithelial cell) and BPH1 (benign prostate hyperplastic epithelia cell) cell lines. Results showed that PC3 cells internalized coated particles with higher efficiency than BPH1 cells. Boyoglu et al. (2013) used SEM analysis to correlate the size of gold nanoparticles with their cellular localization in HEp-2 cells. Results showed that the presence of nanoparticles in the cytosol and nucleus was simply based on their sizes, regardless of the incubation time.

Among label-free techniques, Raman microscopy presents several advantages useful to study nanoparticles uptake. Compared to SEM and TEM analysis, Raman microscopy requires minimum sample preparation and allows *in vitro* and *in vivo* cellular imaging. This methodology was used by Managò et al. (2018) to evaluate the internalization kinetics and intracellular localization of diatomite-based nanoparticles in a lung epidermoid carcinoma cell line. The results indicated the presence of NPs up to 72 h, without damage to cell viability or morphology. Raman microscopy has also been used to study the uptake of metal-based nanoparticles. Using this technique, Chaves et al. (2017) explored the cellular uptake of iron oxide nanoparticles in breast cancer cells. The NPs were totally internalized in cells, displaying a cytoplasmic localization with a direction toward the nucleus after 24 h of incubation.

Label-based techniques include methods that exploit a fluorescent signal emitted by the nanoparticles. This signal can be attributed to an intrinsic property of the NPs (Suzuki et al., 2007; Chen et al., 2014) or to the use of a fluorophore added to the nanoparticles. This modification can be obtained adding the fluorescent tag inside the NPs structure during synthesis or binding the tag to the NPs surface.

The use of this approach to study NPs cellular uptake has several advantages like the ease of use, no need or simple specimen preparation, possibility to analyze live-cells and to perform time-lapse acquisitions. However, fluorescence methods do not allow a quantitative analysis, but only comparison between different experimental conditions (semi-quantitative analysis). This is due to the fact that the signal is not absolute because its intensity depends on the excitation source, the number of fluorophores per NP, the quantum yield of fluorophores or the NP itself, and the sensitivity of the detector (Drasler et al., 2017).

The most used label-based techniques to study NPs cellular uptake are confocal fluorescent microscopy (CFM) and flow cytometry (FC).

Owing to very high resolution, contrast and penetration depth, CFM allows the detection of very low amounts of NPs and to localize them to cellular compartments. Costanzo et al. (2019) used CFM to study the uptake of various types of NPs [liposomes, mesoporous silica NPs, poly(lactide-co-glycolide) NPs, and nanohydrogels] in myoblasts with a high proliferative rate and in myotubes characterized by a low proliferative rate, to correlate proliferative rate with NPs uptake. The results showed that there was a lower uptake in myotubes compared to myoblasts and the inability to penetrate in nucleus by all analyzed NPs in both cell types. Silvestri et al. (2019) evaluated melanin-silica hybrid nanoparticles uptake in two pancreatic cancer cell lines where NPs appeared as punctate small vesicles, indicating an endocytic mechanism of internalization.

The use of flow cytometry to study NPs uptake is widely used, despite the impossibility of discriminating NPs attached on cellular surface from the internalized ones. Jochums et al. (2017), using NPs labeled with the fluorescent dye fluorescein isothiocyanate (FITC), evaluated TiO_2_ NPs uptake in NIH/3T3 and A549 cell lines. The authors observed that NIH/3T3 cells internalized the TiO_2_ NPs more efficiently than A549 cells, confirming the importance of the cell type in the uptake behavior. Flow cytometry can also be used to investigate the internalization pathway of the nanoparticles. Gao et al. (2013) investigated the uptake pathway of bare and IL13-modified nanoparticles using several specific endocytosis inhibitors and flow cytometry. Their results showed that bare NPs were internalized by macropinocytosis while IL13-modified NPs were internalized by a clathrin-mediated pathway, typical of a receptor-mediated uptake.

### NPs Biocompatibility Evaluation

**FIGURE 2:**
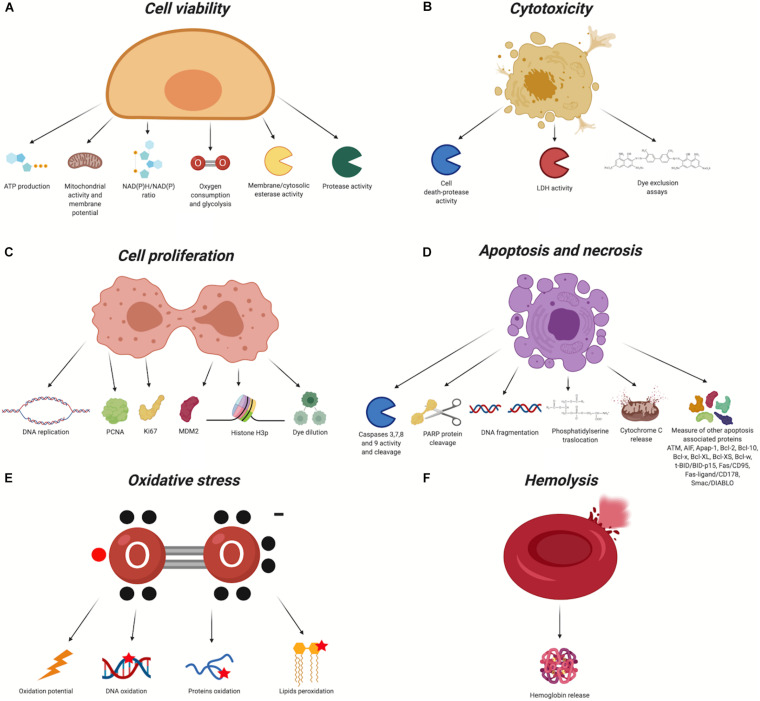
Biocompatibility evaluation assays (Created with BioRender.com). In red the chemical groups that react with the nanomaterial. X, organic/inorganic free chemical groups used to bind the ligands. **(A)** Cell Viability, **(B)** Cytotoxicity, **(C)** Cell Proliferation, **(D)** Apoptosis and necrosis, **(E)** Oxidative stress, **(F)** Hemolysis.

To study NPs biocompatibility, there are several aspects, summarized in Figure 2 that can be evaluated: cell viability (Figure 2A), cytotoxicity (Figure 2B), proliferation (Figure 2C), apoptosis/necrosis (Figure 2D), cellular morphology alteration, oxidative stress (Figure 2E), inflammatory response, and hemotoxicity (Figure 2F). Using different methodologies exploiting commercial kits and/or protocols adapted to the type of analysis it is possible to assess these aspects. Some of the most useful markers/pathways are summarized in Table 2.

**TABLE 2:** Biocompatibility evaluation assays used to asses NPs toxicity.

	Description	Detection method	Info	References
*Cell viability* (evaluation of cells health state)	Measure of ATP concentration	Fluorescence, colorimetry, luminescence	The ATP assay showed very high versatility and ease-to-use in fact it is used for several different types of nanoparticles like high-density lipoprotein, gold, silver and silica. Furthermore, the use of luminescence as ATP quantification method doesn’t show interference with fluorescence/adsorption of nanoparticles	Vetten et al., 2013; Cao et al., 2017; Suganuma et al., 2017; Fehaid and Taniguchi, 2019; Silvestri et al., 2019; Wang et al., 2019
	Evaluation of NAD^+^/NADH ratio	Luminescence, colorimetry, fluorescence	NAD^+^ and NADH are very important molecules for a lot of cellular processes and their levels are related to cell health. The disponible luminescence-based assays can avoid fluorescence/adsorption of NPs	Da Veiga Moreira et al., 2016; Patgiri et al., 2020
	Measure of mitochondrial membrane potential (ΔΨm)	Fluorescence	5,5′,6,6′-Tetrachloro-1,1′,3,3′-tetraethyl-imidacarbocyanine iodide (JC-1) dye accumulates in the mitochondrial membrane depending on membrane potential. The high potential of the inner mitochondrial membrane induces the formation of the dye aggregates. Free dye and aggregates have different fluorescence properties. This shift is used to analyze mitochondria membrane potential	Chen C. et al., 2017; Popov et al., 2020
	Evaluation of membrane/cytosolic esterase activity	Fluorescence	Membrane esterase evaluation to study cell viability is widely used to assess NPs toxicity both with flow cytometry and fluorescence microscopy analysis to obtain semi-quantitive and qualitative data, respectively	Bancos et al., 2012; Grudzinski et al., 2013; Singh and Lamprecht, 2016; Hernandez-Delgadillo et al., 2018; Silvestri et al., 2019
	Measure of oxygen consumption and glycolysis	Fluorescence, Luminescence	The evaluation of extracellular oxygen consumption rates (OCR) is directly related to cell health and mitochondria activity. Furthermore, the evaluation of L-lactate produced by glycolysis is widely used to evaluate cellular health state	Luo et al., 2015; Grahovac et al., 2019; Yun et al., 2019; Zhu et al., 2019; Adeyemi et al., 2020
	Evaluation of live-cell protease activity	Fluorescence	The live-cell protease activity is limited to intact cells and it is evaluated by using a fluorogenic cell-permeant peptide as substrate (Gly-Phe-AFC). It is interesting that live-cell proteases activity decreases when plasmatic membrane loses its integrity and the enzymes are released in culture medium. This feature can help to discriminate alive cells from dead cells and to reduce false positives	Amin et al., 2017; Zhang et al., 2020
*Cytotoxicity* (evaluation of dead cells)	Membrane damage evaluation by dye exclusion	Colorimetry	The evaluation of membrane integrity alteration assessed with dyes (usually Trypan Blu and Erythrosine B) is easy and cheap, but in dye exclusion cytotoxicity assays (i) survive live cells can continue to proliferate and (ii) some dead cells can’t be revealed because they may undergo to an early disintegration	Bejjani et al., 2005; Karlsson et al., 2008, 2013; Fakhimikabir et al., 2018
	Activity evaluation of released lactate dehydrogenase	Colorimetry, Fluorescence, Luminescence	The release of lactate dehydrogenase from cells is one of the most useful marker of cell death. NPs could interfere with enzymatic activity and/or with colorimetric assays	Forest et al., 2016; Chen C. et al., 2017; Gea et al., 2019; Hu et al., 2019
	Activity evaluation of cell-death related proteases	Fluorescence, Luminescence	Cell death-proteases release assay estimates the activity of the intracellular enzymes when they are released after membrane damage. These cell-death proteases have high activity on specific target sequences (like Ala-Ala-Phe-R), different from the other intracellular proteases (like Gly-Phe-R) and for this reason the assay is very specific. Furthermore, the possibility to perform luminescence assay can reduce NPs interference	Gonnissen et al., 2016; Iglesias et al., 2017; Zhang et al., 2017; Gurunathan et al., 2018
*Cell proliferation* (evaluation of mitotic rate)	Evaluation of DNA synthesis	Fluorescence, Colorimetry, Luminescence	The evaluation of cell proliferation through DNA synthesis is performed by incorporation of nucleoside-analog like 5-bromo-20-deoxyuridine (BrdU) in DNA. This simple assay is widely used to assess cell proliferation and genotoxicity in presence of different nanomaterials. Furthermore, BrdU assay can be performed both *in vitro* and *in vivo*	Ruizendaal et al., 2009; Hernández-Ortiz et al., 2012; Al-Qubaisi et al., 2013b; Chen M. et al., 2019; Kutwin et al., 2019
	Dye dilution	Fluorescence	During cell proliferation for each generation the amount of dye in each cell is shared between two cells. The cell proliferation can be monitored by analysis of dye fluorescence reduction	
	Protein cell nuclear antigen activation (PCNA)	Chemiluminescence, Fluorescence, Colorimetry	PCNA is a DNA clamp essential for DNA replication in eukaryotic cells. Its concentration increases during cell proliferation. PCNA has a lifetime of about 20 h and for this reason it could be detected also in non-proliferative cells, causing wrong data about cell proliferation	Khdair et al., 2010; AshaRani et al., 2012; Chairuangkitti et al., 2013; Li Q. et al., 2018; Kutwin et al., 2019
	Ki-67 activation	Chemiluminescence, Fluorescence, Colorimetry	Ki-67 protein concentration increases in nucleus during cell progression in S phase. Ki-67 that is present in all cell cycle steps except in G0 state has a half-life of about 36 h and could be still detected in the first phase of quiescence	Ludwig et al., 2017; Marino et al., 2018
	Minichromosome maintenance protein 2 (MCM-2)	Chemiluminescence, Fluorescence, Colorimetry	MCM2 is a protein involved in the beginning of DNA replication (pre-replication complex) and cell proliferation. MCM2 is highly expressed in early G1, low expressed in S, G2, and M phases, and it is totally absent in G0. Furthermore, MCM2 shows distinct cellular localization in cycling cells and this pattern can be used to evaluate cellular proliferation	Yang et al., 2010
	Phosphohistone H3 (PPH3)	Chemiluminescence, Fluorescence, Colorimetry	Histone-3 is extensively phosphorylated (serine-10 and serine-28) during mitosis and it is widely used to study cell proliferation	Duong Le et al., 2016; Surapaneni et al., 2018; Tang et al., 2018; Brzóska et al., 2019
*Apoptosis and necrosis* (evaluation of apoptotic/necrotic cells)	Evaluation of DNA content	Fluorescence	One of the most used molecules to study DNA content is propidium iodide. This molecule has fluorescent excitation maximum at 495 nm and emission maximum at 630 nm. When PI binds DNA its quantum yield increases of about 20–30 folds and a significative fluorescence red shift (535/615 nm) is observed. PI used at low concentrations (<50 μg/ml) cannot pass through the biological membranes of healthy cells but can penetrate into damaged cells like necrotic or late apoptotic cells, allowing their identification. Furthermore, the use of a permeabilizing that allows PI entry in all cells makes possible to evaluate apoptotic cells by studying cell cycle pattern (due to DNA fragmentation, apoptotic cells have lower DNA content compared to healthy cells)	Kumar et al., 2015; Li et al., 2019b; Silvestri et al., 2019; Yang et al., 2019a
	Evaluation of phosphatidylserine translocation	Fluorescence	To discriminate necrotic cells from apoptotic ones an additional staining is necessary. During early apoptosis process, translocation of phosphatidylserine (PS) from the inner to the outer side (extracellular side) of the plasma membrane can be detected by using Annexin V protein conjugated with several different fluorescent dyes. **Propidium Iodide Annexin V Type of cell** **Negative** Negative Healthy cell **Negative** Positive Early apoptotic cell **Positive** Negative Necrotic cells **Positive** Positive Late apoptotic cells	Kim et al., 2019; Xiao et al., 2019; Zhu et al., 2019; Adeyemi et al., 2020; Ding et al., 2020; Elkeiy et al., 2020
	Measure of nick breaks in DNA	Fluorescence, Colorimetry	The most used assay to evaluate the fragmentation of nuclear DNA in consequence of apoptosis is the TUNEL assay. This assay is based on the use of an enzyme (Terminal Deoxynucleotidyl Transferase) that adds dUTP to the 3’-OH DNA ends. The use of labeled dUTP (with fluorescent or chromogenic dyes) can allow the identification and the quantification of DNA fragments by using microscopy or flow cytometry	Popovtzer et al., 2016; Yu et al., 2017; Phuong et al., 2018; Ding et al., 2020
	Evaluation of PARP protein cleavage	Chemiluminescence, Fluorescence, Colorimetry	Poly-ADP-ribose polymerase (PARP) is widely used to assess apoptosis. In apoptotic cells PARP is a substrate for caspase-3 enzyme. PARP protein (116 kDa) and its cleavage by caspace-3 in apoptotic events (which produces an 85 kDa fragment) is a useful marker to evaluate apoptosis	Kang et al., 2009; Zhang et al., 2013; Al-Shakarchi et al., 2018; Wang et al., 2018; Ahmad N. et al., 2019
	Evaluation of caspases and their cleavage	Fluorescence, Luminescence, Colorimetry, Chemiluminescence	Caspase are very important markers of apoptosis. They are synthesized how pro-enzymes and activated during apoptosis by proteolytic cleavage. There are several different ways to evaluate the active enzymes based on the use of specific antibodies: flow cytometry, western blot or immunohistochemistry. Furthermore, it is possible to evaluate caspases enzymatic activity in cells by using specific substrates trough fluorescence, colorimetry or luminescence both in real-time and in cellular extracts	Baharara et al., 2016; Al-Shakarchi et al., 2018; Blanco et al., 2018; Kalaiarasi et al., 2018; Ahamed et al., 2019; Kang et al., 2009; Baharara et al., 2016; Banerjee et al., 2017; Zhang et al., 2017; Kalaiarasi et al., 2018
	Cytochrome C release	Chemiluminescence, Fluorescence, Colorimetry	The enrichment of cytochrome C in cytoplasm and its decrease in mitochondria can be detected by several techniques, like western blot or immunofluorescence	Al-Qubaisi et al., 2013a
	Measure of other apoptosis associated proteins: ATM, AIF, Apap-1, Bcl-2, Bcl-10, Bcl-x, Bcl-XL, Bcl-XS, Bcl-w, t-BID/BID-p15, Fas/CD95, Fas-ligand/CD178, Smac/DIABLO, p53.	Chemiluminescence, Fluorescence, Colorimetry	There are a lot of proteins involved in apoptosis that can be detected by using specific antibodies	Plackal Adimuriyil George et al., 2018
*Hemotoxicity*	Hemoglobin release assays	Spectrophotometric and naked eye	Hemolysis can be easily evaluated by measuring hemoglobin released from red blood cells by naked eye evaluation (qualitative) or by spectrophotometric analysis at 577 nm (quantitative)	Sanità et al., 2020
*Oxidative stress*	General oxidative stress	Fluorescence, Luminescence	General oxidative stress assays are based on cell-permeable molecules with low or not fluorescence in a reduced state. When these molecules go inside cells they will be oxidized and become fluorescent proportionally to the oxidative potential in cells. In the past two molecules were widely used to evaluate general oxidative stress: 2’,7’-dichlorodihydrofluorescein diacetate (H_2_DCFDA) and dihydroethidium (DHE). Due to several limitations of both molecules like need to serum-free media, low stability, incompatibility with PFA fixing, GFP and RFP and detergents, innovative fluorogenic/luminogenic oxidative stress reagents are product to evaluate oxidative stress	Aranda et al., 2013; Ghosh et al., 2016; Ahamed et al., 2019
	DNA oxidation	Colorimetric (ELISA)	For the evaluation of DNA oxidative stress, analysis of 8-hydroxydeoxyguanosine (8-OHdG) in DNA can be performed. 8-OHdG is a DNA modified base produced by hydroxyl radical attack of guanine in oxidative stress conditions. The 8-OHdG evaluation assay is usually used in combination with others assay to evaluate DNA integrity, like COMET and TUNEL assays.	Ghosh et al., 2016; Kinoda et al., 2016; Ng et al., 2017
	Protein oxidation	Chemiluminescence, Fluorescence, Colorimetry,	Carbonyl groups (aldehydes and ketones) are usually specific markers of proteins oxidation. The amount of carbonyl groups can be detected by using 2,4-Dinitrophenylhydrazine (DNPH) that reacting with the carbonyl groups on proteins produces a DNP-tag detectable by western blot or ELISA. Furthermore, there are also DNPH-modified molecules that can be detected by colorimetric or fluorescent methods	Arya et al., 2016; Jayaram et al., 2017
	Lipid peroxidation	Colorimetry (ELISA), Fluorescence	During oxidative stress, lipid peroxidation produces reactive aldehydes such as the mutagenic compound malondialdehyde (MDA) and the toxic compound 4-hydroxynonenal (4-HNE). MDA molecule is the most used marker to evaluate lipid peroxidation, because it reacts with thiobarbituric acid (TBA) to produce an MDA-TBA adduct that can be easily detected by colorimetric, fluorimetric and ELISA assays	Gaharwar et al., 2017; Ahamed et al., 2019

*^*a*^The use of tetrazolium salt based assays (like MTT, MTS,S and WST-1) or crystal violet can be affected by NPs absorbance properties (Almutary and Sanderson, 2016) resulting in high toxicity overestimation, for this reason they are not added to this table.*

## NPs Surface Modification to Enhance Biocompatibility and Uptake

The physiochemical properties of NPs, like shape, size, charge, material and surface chemical groups, influence their toxicity and uptake efficiency. Some of these, such as surface charge and chemical groups, can be easily modified by surface modification.

### Biocompatibility and Immune Escape

The functionalization of the NPs surface to enhance biocompatibility can be exploited by using different molecules; among these PEG is one of the most used for *in vitro* and *in vivo* applications. Kostiv et al. (2017) showed how the addition of PEG on the surface of Fe_3_O_4_ and SiO_2_ NPs increased the biocompatibility when PEGylated NPs were used at high concentrations (200 μg/mL) with murine neural stem cells, unlike bare Fe_3_O_4_&SiO_2_ that caused a viability reduction of about 50% already at a dose of 20 μg/mL. This study is a clear example of the high convenient properties of PEG to increase NPs biocompatibility. Furthermore, the presence of PEG on the NPs surface also improves the hemocompatibility, as observed with chromium-doped zinc gallate and diatomite based NPs (Terracciano et al., 2015; Jiang et al., 2019).

To reduce the toxicity of NPs, dextran is a widely used to modify nanoparticles. Dextran is a complex branched polysaccharide usually exploited to modified iron-oxide NPs surface. de Oliveira et al. (2017) showed how the addition of dextran to iron-oxide NPs increased biocompatibility in zebrafish larvae; in particular, the treatment with dextran-coated NPs did not determine any significant mortality or changes in the hatching rate of the larvae. The toxicity of dextran modified iron-oxide NPs was also investigated by Balas et al. (2017) on Jurkat cells. The authors observed low toxicity and small effects on membrane integrity up to 72 h of incubation. The use of dextran with iron-oxide nanoparticles to improve biocompatibility was also studied using primary cells. Wu et al. (2018) demonstrated that dextran-NPs had no significant effects on cell viability and apoptosis on human primary monocytes cells.

A further oligosaccharide generally used to enhance NPs biocompatibility is chitosan, as reported by Shukla et al. (2015) and Peng et al. (2017). Shukla et al. (2015) observed a decrease of toxicity in three different cell lines when chitosan-NPs were compared to iron-oxide NPs. Peng et al. (2017) used chitosan to modify silver NPs. Results showed that chitosan-coated silver nanoparticles had higher biocompatibility when compared with silver nanoparticles without surface modification in human fibroblast cells.

A more complex NPs surface modification as way to enhance biocompatibility, stability and dispersity was been reported by Han et al. (2016). The study demonstrated that encapsulation of mesoporous silica NPs using a lipid bilayer is a useful way to improve biocompatibility and hemocompatibility.

Besides the increase of biocompatibility, NPs surface modification is a very important tool for the modulation of the body’s immune response against the particles (Qie et al., 2016; Boraschi et al., 2017; Visalakshan et al., 2019). NPs, after the injection in blood flow, interact with a lot of aspecific proteins like opsonin, complement proteins, immunoglobulins, fibronectin, and apolipoproteins (protein corona) and this interaction can modify NPs behavior and can trigger an immune response (Barbero et al., 2017). The NPs surface modification can be exploited to promote their escape from the immune system and to increase their half-life in blood by reducing the clearance due to macrophages of the mononuclear phagocyte system (MPS). Conversely, in the next-generation vaccines, the NPs surface modification can trigger the immune response toward a specific antigen (Ahmad S. et al., 2019; Cappellano et al., 2019; Gu et al., 2019).

Escape from the immune system can be obtained through the modification of NPs surface with different types of molecules that make NPs “invisible” to the immune system cells. For this purpose, the most used compounds are hydrophilic polymers that bind water molecules producing a shield on NPs surface. This layer of water reduces the interaction with opsonin and/or macrophages receptors (Pinzaru et al., 2018). This strategy can increase NPs circulation time and can reduce the clearance (Abdollah et al., 2018).

### Uptake

It is well known that the cellular uptake of NPs is influenced by the physicochemical properties of NPs, such as its composition, size, shape, surface charge, surface functionalization, and surface hydrophobicity/hydrophilicity. When nanoparticles interact with the constituents of the plasma membrane, they are mainly taken up by cells by endocytosis, which is commonly classified in phagocytosis (macrophages) and pinocytosis (all cellular types). The latter can be distinguished in clathrin-dependent endocytosis, caveolae-dependent endocytosis, macropinocytosis, and clathrin, and caveolae-independent endocytosis. The modification of the physicochemical properties of NPs surface can be exploited to enhance cellular uptake.

#### Passive Uptake

PEG, usually tested to enhance biocompatibility, can also be explored to increase NPs uptake. The PEG monomer is about 0.35 nm in length with a molecular weight of about 2 kDa, and can be synthesized at different lengths. This molecule reduces aggregation and increases NPs stability in biological fluids (Desai et al., 2016; Harrison et al., 2016). Furthermore, PEGylation of NPs determines a decrease of the interaction with not-specific proteins resulting in a “stealth” effect able to enhance PEG-NPs circulation time and to reduce phagocytosis (Vonarbourg et al., 2006). This increased stability of PEG-NPs is directly related to higher cellular uptake if compared with bare NPs that can aggregate in biological environment and/or to be phagocytized by immune system cells.

As reported by Cu and Saltzman (2009), the use of PEG can effectively change the behavior of NPs in relation to its delivery in tissues. The addition of PEG of different sizes (2.5, 5, and 10 kDa) on poly(lactic-co-glycolic) acid (PLGA) on the NPs surface can enhance particle diffusion by up to 10-fold into the cervical mucus and the binding to mucin proteins, depending on the PEG density and size. Furthermore, the conjugation with PEG results in a shift in the charge of PLGA NPs, from −45 to + 8 mV, with the NPs size increase related to the efficiency of PEG coverage. Cruje and Chithrani (2014) showed that NPs properties can be affected by the length of PEG molecule and by the surface functionalization density (PEG molecules/nm^2^). In particular, when longer PEG molecules and a high density were used to functionalize gold NPs, a reduction in the non-specific protein adsorption was observed. However, this kind of modification resulted in a decrease in the uptake of NPs in all cell lines tested.

Other molecules used to modify NPs surface involve changes in the superficial charge. Generally, these consist of amino ending molecules (R-NH_2_) that at physiological pH (about 7.4) are positively charged. Rancan et al. (2012) demonstrated how the surface charge of silica nanoparticles (SiO_2_) affects the cellular uptake of NPs in the HaCaT cell line and in skins explants. Negatively charged SiO_2_ NPs showed lower uptake levels compared to positive 3-aminopropyl-trimethoxysiliane (APS) modified NPs. Conversely, Liu et al. (2011) reported that polystyrene-modified NPs with -NH_2_ groups on the surface enhanced toxicity, related to the high reactivity of the amino groups. The higher uptake and toxicity of positively charged NPs, compared to negatively charged NPs, has also been demonstrated by Bannunah et al. (2014) in an intestinal epithelial cell model.

Furthermore, the superficial charge of NPs can be selectively modified to modulate the cellular uptake. Zwitterionic ligands, like carboxybetaines and sulfobetaines, show a variety of the positively and negatively charged groups allowing the modulation of charge densities to optimize solubility and to avoid the interactions of the protein corona, making NPs highly stable in biological fluids. This modification reduces non-targeted uptake and opsonization of modified-NPs, and increases their accumulation in target tissues (e.g. cancer cells) (Breus et al., 2009; Muro et al., 2010; Zhang et al., 2011). An example of the zwitterionic ligands useful to enhance NPs uptake is reported by Drijvers et al. (2019). In this work, silica coated CdSe/CdS quantum dots were biofunctionalized with PEG and with sulfobetaines in order to evaluate the impact of these modifications on the NPs cellular uptake. The authors showed the differences in the cellular internalization and, in particular, they revealed that the uptake of the zwitterion-modified NPs happened more easily compared to PEG-modified NPs in HeLa cells.

In Mosquera et al. (2018) gold-NPs were modified with pyranine (a negatively charged dye) that reduced the NPs uptake. This behavior can be reversed through the addition of a positively charged molecular cage that neutralizes the negative charge of gold NPs and allows cellular uptake. The key role of the surface charge of NPs in cellular uptake efficiency was also demonstrated by Jeon et al. (2018), where the surface of fluorophore-conjugated polystyrene nanoparticles (f-PLNPs) was modified with different types of functional groups (acetyl, zwitterionic, carboxyl) and molecules (guanidinium, polyethylene glycol, sulfonic acid). The nanoparticles were incubated with THP-1 cells (phagocytic) or A549 cells (non-phagocytic). Results showed a NPs superficial charge-dependent uptake by both cell lines; in particular, an increasing trend in internalization was observed in positively charged modified-NPs and this correlation was stronger in the THP-1 cells compared to A549 cells.

Another strategy widely explored to increase NPs uptake is based on the use of cell penetrating peptides (CPPs). These molecules are composed of a specific aminoacidic sequence, usually polycationic or amphipathic structures, that enhance NPs uptake. Asai et al. (2014) used a CPP derived from protamine to modified lipid-based NPs for the efficient delivery of siRNA. This modification enhanced internalization of siRNA in B16F10 cells. Feiner-Gracia et al. (2018) exploited the penetrating capability of Tat peptide to functionalize PLGA NPs surface. They observed that while bare NPs did not enter HeLa cells, when Tat peptide was added (Tat-NPs), cellular uptake was detected.

Due to the importance of enhancing the uptake of NPs, research into alternative molecules that bind on the NPs surface is very active. Yang et al. (2018) reported the use of phosphatidylcholine (PC) modified with different alkyl chain lengths (from C12 to C18), to increase lipid-PLGA hybrid NPs internalization. Even if PC had little effect on NPs stability and physicochemical properties, results showed an enhanced cellular uptake of hybrid nanoparticles in HepG2 or A549 cells. Furthermore, the PC-modified NPs uptake increased proportionately to the length of PC alkyl chain.

#### Active Uptake

**FIGURE 3:**
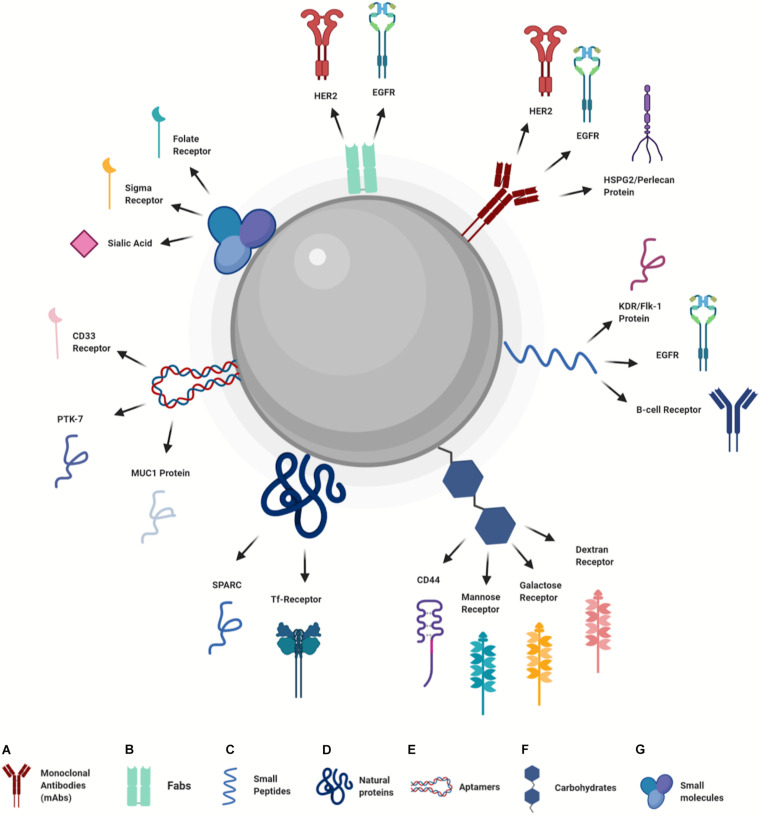
Design of nanoparticles for active uptake (Created with BioRender.com). **(A)** Monoclonal Antibodies, **(B)** Fabs, **(C)** Small Peptides, **(D)** Natural Proteins, **(E)** Aptamers, **(F)** Carbohydrates, **(G)** Small molecules.

In recent years, surface modification of NPs was mainly aimed at active targeting and cellular uptake by exploiting the specific interactions of NPs surface ligands with a wide range of receptors overexpressed in cancer cells (Salahpour Anarjan, 2019). The molecules useful to perform NPs active uptake include several main categories, among these the most used are antibodies, small peptides, proteins, aptamers, carbohydrates, and small molecules (Figure 3). During the conjugation process, usually performed by covalent interactions, these molecules are linked to the NPs surface in order to preserve their ability to bind the target receptors.

Monoclonal antibodies (mAbs) used to perform active uptake of NPs are extensively studied due to their high stability, specificity and binding ability (Figure 3A). Fathian kolahkaj et al. (2019) reported a very efficient uptake of PLGA NPs modified with an anti-HER2 mAb. Uptake evaluation in HER2 positive (MDA-MB-453) and negative (MCF-7 and BT-20) cells demonstrated higher internalization levels in a HER2 positive cell line, compared to the negative ones. Another example related to the use of targeted uptake with the anti-HER2 antibody was reported by Wu et al. (2019). In this work, boron-containing gold NPs were bioconjugated with an anti-HER2 antibody and their uptake was studied both *in vitro* and *in vivo*. Results showed an elevated selective uptake in N87 human gastric cancer cells of mAb-gold NPs when compared to bare NPs. *In vivo* experiments indicated accumulation of mAb-gold NPs in tumor sites and a reduction of non-specific uptake at others anatomical districts. Another receptor widely used to perform active uptake in cancer cells is the epidermal growth factor receptor (EGFR). As reported by McDaid et al. (2019), the functionalization of PLGA NPs surface by using a therapeutic mAb against EGFR (Cetuximab) enhanced the NPs uptake in an *in vivo* model. A further example of mAb modified NPs to improve cellular internalization was reported by Khanna et al. (2019), where PLGA nanoparticles were functionalized with a mAb against heparan sulfate proteoglycan 2 (HSPG2) protein, a surface receptor highly expressed in triple negative breast cancer (TNBC) that binds growth factors such as VEGF-A and FGF-2, acting as “co-receptor.” Even in this study the presence of mAb on the NPs surface increased specific cellular uptake in Luciferase-expressing MDA-MB-231-LM2 cells.

Antibodies are very useful molecules to enhance specific NPs uptake, but they have high molecular weight (about 150 kDa), and this could be an issue in bioconjugation process, above all with smaller NPs (<10 nm). The use of the antibody antigen-binding fragments (Fabs) to perform NPs active uptake is a viable alternative, as reported by Houdaihed et al. (2020) (Figure 3B). To target breast cancer cells, the PEG-PLGA NPs were loaded with paclitaxel and everolimus and the NPs were coated with anti-HER2 and anti-EGFR Fabs. Results showed higher uptake levels in HER2 and EGFR positive cell lines (SKBR3) compared to negative or low EGFR expressing cell lines (MCF-7 and MDA-MB- 436).

A viable alternative to the use of antibodies and Fabs to modify NPs surface is represented by peptides that bind with high affinity to specific receptors (Figure 3C). Indeed, specific peptides can be obtained by the screening of phage libraries and by the isolation of binding sequences from proteins using three-dimensional (3D) structural analysis. A small peptide (GE11) with high affinity for EGFR was used by Li et al. (2019a) to modify nanomicelle containing evodiamine (GE11-Evo-NPs) in order to enhance drug uptake into cancer cells. Results obtained *in vivo* showed that GE11-Evo-NPs allow higher evodiamine concentration in tumor after intravenous administration, compared to the drug alone. Furthermore, the accumulation of GE11-Evo-NPs in tumor masses *in vivo* was higher than Evo-NPs. Another study where a peptide was used to enhance active NPs accumulation is reported by Qian Q. et al. (2019). In this study, hybrid chitosan/poly(N-isopropylacrylamide) NPs functionalized with K237 peptide (that binds KDR/Flk-1 receptor) were described. The modified NPs showed higher uptake in the MDA-MB-231 cell line overexpressing KDR/Flk-1 compared to K237 free NPs and to L929 cells that do not express the KDR/fLK-1 receptor. The use of peptides was also explored to perform a personalized therapy approach. Martucci et al. (2016) showed that the surface functionalization of diatomite-based NPs with an idiotype-specific peptide (Id-peptide), which recognizes hypervariable region of immunoglobulin B-cell receptor, enhanced Id-NPs uptake in specific myeloma cells of threefold compared to nonspecific cells. Furthermore, same results were obtained if a random control peptide was used.

The use of natural proteins that interact with cancer cell receptors has been widely investigated, as reported in several recent studies (Figure 3D). Scheeren et al. (2020) used transferrin (Tf) to functionalize the surface of doxorubicin-loaded PLGA NPs. The interaction between transferrin and Tf receptors (Tf-R), highly expressed in cancer cells, was exploited to enhance uptake and drug release. Results showed that Tf-PLGA@DOX NPs greatly reduced viability of human epithelial cervical cancer cells HeLa (Tf-R positive), when compared to immortalized HaCaT keratinocytes with low Tf-R expression. Another protein used to perform NPs active delivery is human serum albumin (HSA) that interacts with tumor-associated protein SPARC. Sanità et al. (2020) modified hybrid melanin-silica-silver nanoparticles (MelaSil_Ag) surface using HSA to enhance cellular uptake in breast cancer cells. Results showed that MelaSil_Ag-HSA NPs were mostly internalized by SPARC positive cell line (HS578T) compared to SPARC negative cells (MCF10a).

Furthermore, other molecules are currently being explored to modify NPs surface and to perform active uptake. Among these, aptamers represent a useful molecule since are cheap and easy to synthetize (Figure 3E). Aptamers are short nucleic acid sequences (dsDNA, ssDNA or RNA) with specific 3D structure that can bind a molecular target in cancer cells with high specificity and affinity. Mie et al. (2019) reported the conjugation of the aptamer S-MUC-1 (able to bind MUC1 protein) on paclitaxel-loaded protein nanoparticles. Incubation of aptamer-modified and bare NPs with MCF-7 cells (that overexpress MUC-1 protein) showed higher uptake levels of aptamer-functionalized NPs when compared to the bare NPs. Further examples of aptamers as ligand for active uptake are reported by Gui et al. (2019) and Yang et al. (2019b). Yang et al. (2019b) modified the surface of mesoporous silica nanoparticles (MSNs) with Sgc8 aptamer able to bind protein tyrosine kinase-7 (PTK-7) on human acute T lymphocyte leukemia cells. Sgc8-MSNs NPs were also loaded with DOX, in order to enhance drug uptake in leukemia cells. The results obtained with two different cell lines, CCRF-CEM with high expression of PTK-7 and Ramos cells with no expression of PTK-7, showed a high uptake level of Sgc8-MSNs in CCRF-CEM cells compared to Ramos cells. Furthermore, no differences in NPs uptake either cell lines was observed using bare MSNs. Gui et al. (2019) used an aptamer designed to bind CD133 receptor on osteosarcoma cells in order to enhance the uptake of lipid NPs loaded with all-trans retinoic acid (ATRA). Results showed high internalization of aptamer-modified NPs in CD133 positive cells compared to the negative ones, confirming the CD133 mediated targeting.

Exploitation of simple molecules to enhance active uptake also includes the use of carbohydrates (Figure 3F). Among these, one of the most studied is hyaluronic acid (HA). This molecule enhances the NPs uptake through its interaction with CD44 protein. Li et al. (2020) recently demonstrated that HA can be easily used to biofunctionalized carbon dots loaded with doxorubicin to enhance drug uptake in CD44 overexpressing cells (4T1). Results obtained by competitive assay performed with free HA showed that the HA-modified NPs were internalized through the binding of HA-modified NPs with the CD44 receptor. Furthermore, *in vivo* experiments showed that the enhanced accumulation into tumor tissue was confirmed. The use of carbohydrates was also explored for targeted gene-delivery in macrophages by Chen et al. (2020). PLGA-PEG NPs decorated with various carbohydrates (mannose, galactose and dextran) and loaded with eGFP (enhanced Green Fluorescent Protein) mRNA and GFP (Green Fluorescent Protein) plasmid DNA (used as reporters) was developed. The results showed that carbohydrates on the NPs surface, especially mannose and dextran, improved the active uptake of carbohydrate-modified-NPs in the Raw 264.7 murine macrophage cell line, that express specific receptors for mannose and dextran.

In addition to the molecules already mentioned, there are other small molecules useful to perform NPs active uptake (Figure 3G). Khan et al. (2020) used folate (FA) to functionalize chitosan-lipid hybrid NPs in order to increase uptake through the interaction of FA with folate receptor (FR), highly expressed on cancer cells. Results showed higher uptake of FA-conjugated NPs in an ovarian cancer cell line (SK-OV-3) when compared to bare NPs. Similar results were obtained with the 3D spheroid cell model, with a 2.4-fold higher uptake of FA conjugated NPs compared to uptake by the control group. Other simple molecules used to perform NPs active uptake are anisamide (AA) and phenylboronic acid (PBA) able to bind sigma receptors and sialic acid (SA), respectively. These molecules were successfully investigated by Qian X. et al. (2019) and Ramzy et al. (2020). Ramzy et al., functionalized the surface polymeric NPs loaded with thymoquinone (TQ) by using AA, in order to enhance uptake and drug accumulation in colon cancer cells. Researchers used three colon cancer cell lines, HT-29 (overexpressing sigma receptor), HCT-116, and Caco-2, to evaluate the toxicity of AA-TQ-NPs. Results showed higher toxicity of AA-TQ-NPs in HT-29 cells, compared to the other two cell lines. Furthermore, bare TQ-NPs showed lower toxicity in HT-29 cells compared to AA-functionalized NPs. These results are due to the active uptake of AA-modified NPs after interaction with the sigma receptor. The use of PBA was described by Qian et al. with soy protein-based NPs. The high affinity of PBA for SA, which is overexpressed in tumor cells, was exploited to enhance the uptake of PBA-modified NPs in cell lines with different SA expression. The results showed a high uptake level of PBA-NPs in HepG2 cells (SA positive) when compared to SH-SY5Y cells (SA negative).

## Discussion

In the last years, the development of nanoparticles functionalization strategies is considerably grown, due to potential applications of NPs in nanomedicine. Surface modification is evolved from the use of simple molecules (PEG), to the aim to decrease toxicity, clearance and immune response, to more specific and complex ligands, in order to increase specificity and efficacy. The evolution of the NPs surface bioconjugation went hand in hand with the identification of specific cellular targets. The possibility to tune the NPs physico-chemical properties in combination with the knowledge of cancer biology makes possible the use of these nano-platforms in biomedical applications, both for therapy and diagnostic, providing a great contribution to the advance of nanomedicine.

Uptake and biocompatibility are two of the most important features of a usable nanoplatform for medical applications, and overcoming issues related to these two aspects is the first goal in the development of NPs. For this reason, the study of toxicity and cellular uptake is the first step during the biological assessment of NPs.

The NPs surface modification is a powerful instrument to enhance uptake and biocompatibility, as confirmed by the vast amount of scientific papers focused on this topic. These studies demonstrate that the conjugation of molecules on the NPs surface can effectively enhance biocompatibility both *in vivo* and *in vitro*, due to the modification of surface charge and to the inactivation of reactive chemical groups that can affect cellular membrane stability. Moreover, the addition of specific molecules can also enhance NPs passive and active uptake, reducing systemic toxicity *in vivo* and allowing high precision therapy and/or diagnosis. The binding of molecules on the NP surface can be obtained by covalent and non-covalent approaches. The former is widely used to bind proteins, antibodies, aptamers and peptides exploited to enhance uptake and to perform active targeting, while non-covalent interactions are generally used for loading of drugs and for all molecules that must be released in the cells.

The protocols for the conjugation of molecules on NPs surface depend on the nanomaterial used and on the available functional groups, for this reason the use of linker molecules or the modification of NPs and/or ligands in order to obtain a stable conjugation could be necessary. Furthermore, some molecules used to functionalize NPs have high molecular weight due to the overall size of the proteins and the use of a spacer (e.g., PEG_(n__)_) could be necessary to stave off the ligand from NP surface. The use of spacers also has an effect on conjugation density. In fact, in order to bind a sufficient amount of molecules to the NPs it is necessary to reduce steric hindrance. As reported in numerous studies, the conjugation density is a very important parameter that can affect the behavior of NPs. Moreover, it is important to note that the conjugation of some molecules, usually used to enhance uptake, can also increase the NPs biocompatibility.

Due to the high variability of nanomaterials and ligands to modify the NPs surface, it is not possible to follow specific guidelines to functionalize NPs. It is necessary take in account that: (I) the addition of big size molecules could change the size of the NPs, thus influencing uptake; (II) some molecules used for active targeting could change their 3D structure during the functionalization protocol and to lose the ability to bind target molecules; (III) the conjugation with non-covalent bonds could result in an unstable surface modification due to the influence of environmental conditions like pH and ionic strength; (IV) the conjugation density and the orientation of ligands on NPs surface is a key parameter to enhance uptake; (V) the reduction of toxicity could be due to a reduction in NPs uptake; and (VI) positively charged NPs are usually better internalized by cells.

In conclusion, through the handling of surface characteristics, the nanoparticles can be transformed in smart platforms, containing therapeutic and imaging agents as well as stealth property, delivering drugs to specific tissues and providing controlled release therapy. This targeted and sustained drug delivery decreases the drug related toxicity and the frequency of treatments. Nanoparticles have proven useful in the treatment of cancer, and many other diseases, also providing advancement in diagnostic and theranostic applications.

## Author Contributions

GS and AL designed the review. All authors wrote, read and approved the manuscript.

## Conflict of Interest

The authors declare that the research was conducted in the absence of any commercial or financial relationships that could be construed as a potential conflict of interest.
